# Cell Immobilization for Erythritol Production

**DOI:** 10.3390/jof8121286

**Published:** 2022-12-08

**Authors:** María Hijosa-Valsero, Ana I. Paniagua-García, Rebeca Díez-Antolínez

**Affiliations:** Centro de Innovación en Bioproductos Agroalimentarios (CIBAG), Instituto Tecnológico Agrario de Castilla y León (ITACyL), Polígono Agroindustrial del Órbigo p. 2–6, Villarejo de Órbigo, E-24358 León, Spain

**Keywords:** immobilization, erythritol, winery surplus, repeated batches, fermentation

## Abstract

Nowadays, commercial erythritol synthesis is performed by free-cell fermentation with fungi in liquid media containing high concentrations of pure carbon sources. Alternative fermentation techniques, such as cell immobilization, could imply an economic and energetic improvement for erythritol-producing factories. The present work describes, for the first time, the feasibility of achieving cell immobilization during erythritol production. Cells of the fungus *Moniliella pollinis* were successfully immobilized on a cotton cloth which was placed inside a 2-L bioreactor, where they were fed with red grape must supplemented with yeast extract. They produced 47.03 ± 6.16 g/L erythritol in 96 h (yield 0.18 ± 0.04 g/g) over four consecutive fermentation batches. The immobilized cells remained stable and operative during a 456 h period. The erythritol concentration attained was similar (*p* > 0.05; Tukey HSD test) to the reference value obtained with the use of free cells (41.88 ± 5.18 g/L erythritol) under the same fermentation conditions. The comparable results observed for free and immobilized cells evidences the efficiency of the immobilization system. Therefore, the proposed method for erythritol bioproduction eliminates the need for the continuous preparation of fungal inocula before each fermentation batch, thus reducing the costs of the reagents and energy.

## 1. Introduction

Erythritol is a four-carbon polyol that is used as a sweetener, flavor enhancer, humectant, stabilizer and thickener in foods [1,2]. The main advantages of erythritol are its low glycemic response (suitable for diabetics), non-cariogenic effect, low calorie content, natural origin and good digestibility [1]. Erythritol is classified as GRAS (Generally Recognized as Safe) by the US Food and Drug Administration [3], although the European Food Safety Authority recommends not exceeding a daily erythritol intake of 0.71–0.78 g/kg body weight to avoid laxation effects [4]. This polyol also holds a potential as platform chemical for the synthesis of butadiene, 1,4-butanediol, 2,5-dihydrofuran and tetrahydrofuran [5].

Commercial erythritol production is chiefly performed via the microbial transformation of liquid media containing high concentrations of pure carbon sources, such as sucrose, glucose, fructose or glycerol [6,7]. These carbon sources are converted into erythritol by various fungal strains of the genera *Candida*, *Yarrowia*, *Moniliella* or *Pseudozyma* by means of the pentose phosphate pathway when an excess of sugar, glycerol or salts in the extracellular medium provokes a high level of osmotic pressure on the microorganism [5,8]. Theoretically, 1 mol hexose yields 1 mol erythritol, whereas 3 mol glycerol produces 2 mol erythritol [5]. In this biological process, the C/N ratio plays a major role in triggering erythritol synthesis, and optimal values of C/N = 150 have been suggested for *Y. lipolytica* working with pure glycerol [9], but this ratio should be adjusted for each feedstock and fungal strain to maximize the product generation.

Although some bacteria such as *Fructilactobacillus florum* or *Leuconostoc oenos* can produce erythritol at low concentrations [10,11], fungal strains are preferred for erythritol bulk synthesis due to their higher productivity and proven safety [12,13,14]. In fact, the legislation of the European Union mentions, specifically, the use of *Moniliella pollinis* and *M. megachiliensis* for erythritol production for food applications [15].

Erythritol production from alternative substrates—such as in agriculture, food and industrial by-products and surplus—has attracted much attention lately in order to reduce the reagent costs related to the use of pure sugars or pure glycerol as feedstocks [16,17,18,19,20]. For instance, sugarcane molasses, beet molasses, surplus grape must and crude glycerol have been considered for erythritol biosynthesis [16,18,19]. Additionally, certain process innovations have been proposed, including the use of cellular debris from the erythritol-producing fungi as a nitrogen source for further fermentation batches [21], or the employment of solid-state fermentation (instead of submerged fermentation) for the conversion of solid vegetal biomass, such as peanut-sesame wastes or distillers grains [22,23]. In the latter case, it has been demonstrated that the species *Yarrowia lipolytica* can attach to these solids and serve as a seed for repeated fermentation batches [22].

On the other hand, cell immobilization techniques have been applied successfully to other bioprocesses, which offers advantages in terms of its stability, and it allows for the reuse of the biocatalyst [24,25,26]. Cell immobilization consists of keeping a microorganism inside or on the surface of a supporting material in the long term in such a way that it can grow and reproduce normally while it interacts with the medium and performs its expected metabolic activities of industrial interest. Immobilized microorganisms can be easily recovered, recycled and employed for various consecutive fermentation batches. Moreover, there is no need to prepare new microbial seeds to inoculate each of the batches because the microorganisms are already present (immobilized) in/on the supporting material, and they can colonize the new fermentation broth. To the best of our knowledge, there is no available information on cell immobilization for erythritol production in conventional fermentations (i.e., submerged fermentations with liquid feedstocks). Therefore, an advancement in this regard would provide an economic and energetic improvement for erythritol-producing factories.

The present work aims to assess erythritol production with a cell-immobilization system using surplus grape must as a feedstock. Firstly, five different fungal strains were compared to select the most effective one for the erythritol synthesis from the grape must. The most adequate C/N ratio was assessed by optimizing the yeast extract addition (nitrogen source) to the grape must (carbon source composed of hexoses). As yeast extract is an expensive reagent, the partial replacement of yeast extract by (NH_4_)_2_SO_4_ was also tested for cost-reduction purposes by contrasting different combinations of both of the nitrogen sources. Finally, the fungal cells were immobilized on a cotton cloth which was placed inside a bioreactor in order to determine their ability and stability to produce erythritol during various consecutive fermentation batches.

## 2. Materials and Methods

### 2.1. Microbial Strains

The strains *Moniliella madida* CBS 240.79 and *M. megachiliensis* CBS 567.85 were purchased from Westerdijk Fungal Biodiversity Institute (Utrecht, The Netherlands), *M. pollinis* MUCL 40570 was provided by BCCM (Louvain-la-Neuve, Belgium), whereas *M. acetoabutens* DSM 3551 and *M. suaveolens* var. *nigra* DSM 2552 were obtained from DSMZ (Braunschweig, Germany), and they were used as lyophilized cultures in all of the cases. The strains were reactivated by growing them in a 100-mL Erlenmeyer flask containing 50 mL of a liquid medium with 20 g/L glucose (Sigma-Aldrich Chemie GmbH, Steinheim, Germany), 5 g/L yeast extract (Fluka Analytical—Sigma-Aldrich Chemie GmbH, Steinheim, Germany) and 10 g/L peptone (Biolife Italiana Srl, Milan, Italy). The fungi were incubated at 30 °C and 150 rpm under aerobic conditions in an Infors HT Ecotron orbital shaker (Infors AG, Bottmingen, Switzerland) for 72 h until a cell density of about 5 × 10^7^ cells/mL was attained. Then, 750 μL of this fungal suspension were placed in a cryogenic vial, and they were mixed with 200 μL of glycerol:water (80:20, *v*/*v*) before being preserved at −80 °C as microbial stock solutions.

For the inocula preparation, a loopful of the cryopreserved sample was seeded into the same liquid medium and under the same conditions described above, and it was incubated for 48–72 h until there were approximately 5 × 10^7^ cells/mL.

### 2.2. Description of Grape Must

The red grape must (variety Garnacha) was collected from the Oenological Station of Castile and Leon, ITACyL (Rueda, Spain), in September 2019. It contained 125 g/L glucose, 119 g/L fructose (i.e., 244 g/L total sugars), 0.34 g/L total Kjeldahl nitrogen and 0.73 g/L total phenolic compounds. The other components of the grape must can be found elsewhere [16].

### 2.3. Free-Cell Preliminary Tests in Flask Experiments

Small-scale fermentation tests were performed in 250-mL Erlenmeyer flasks containing 25 mL of grape-must broth. This medium was prepared by supplementing the grape must with a nitrogen source, adjusting its pH to 5.5 with an aqueous solution of NaOH (50% *w*/*w*) and autoclaving it at 120 °C for 15 min. After it was cooled, the medium was inoculated with 3% (*v*/*v*) of the appropriate fungal strain (i.e., 0.773 mL of the inoculum described in Section 2.1), the flask was capped with a polyurethane foam stopper (50 × 50 mm; Fisherbrand, Fisher Scientific SL, Madrid, Spain), and it was incubated for 120 h at 30 °C and 200 rpm under aerobic conditions in an orbital shaker Infors HT Minitron (Infors AG, Bottmingen, Switzerland).

In the first place, the five fungal strains were compared to select the most suitable one for the erythritol production. The grape must was supplemented with 6.7 g/L yeast extract according to a non-optimized standard value from previous works [16], and it was processed and fermented in Erlenmeyer flasks. This experiment was performed in triplicate.

Secondly, the effect of the C/N ratio on erythritol production was studied. The yeast extract—with a total nitrogen (TN) content of 11%—was used as model nitrogen source. The grape must was supplemented with 0, 3, 6, 9, 12 or 15 g/L yeast extract, and it was inoculated with *M. pollinis* MUCL 40570 and incubated in Erlenmeyer flasks. An optimal value for the yeast extract concentration was determined mathematically, and then, it was validated experimentally with a 120 h fermentation tests where the samples were taken every 24 h to assess the temporal evolution of the process. These experiments were performed in duplicate.

Thirdly, after establishing the optimal yeast extract concentration at 6.88 g/L (equivalent to 0.76 g/L TN), the replacement of the yeast extract with a cheaper nitrogen source—ammonium sulphate (Sigma-Aldrich Chemie GmbH, Steinheim, Germany) with a TN content of 21.2%—was assessed. Therefore, several mixtures of yeast extract and ammonium sulphate, whose TN sum was always equivalent to 0.76 g/L, were tested for the fermentation of the grape must with *M. pollinis* MUCL 40570 in Erlenmeyer flasks for 120 h. Table 1 shows the different nitrogen combinations employed. The most adequate conditions were further tested in another 120 h fermentation process, which was monitored every 24 h. These experiments were performed in duplicate.

In all of the cases, the fermentation broths were sampled and analyzed at the beginning of the fermentation process (just after inoculation) to monitor their initial sugar content (Table S1, Supplementary Material).

### 2.4. Free-Cell Batch Fermentation in the Bioreactor

The grape must was supplemented with 6.88 g/L yeast extract. Its pH was adjusted to 5.5 with an aqueous solution of NaOH (50% *w*/*w*), and it was autoclaved at 121 °C for 15 min. After cooling, a sample volume of 1.2 L was placed in a 2-L glass jar bioreactor (B. Braun Biotech International, Melsungen, Germany) which was provided with a baffle. The broth was seeded with 3% (*v*/*v*) of an inoculum of *M. pollinis* MUCL 40570 (the initial cell concentration in the bioreactor was 2.33 ± 0.37 × 10^6^ cells/mL), and it was incubated at 30 °C and 250 rpm for 96 h, with an aeration of 3 L/min (2.5 vvm) which was produced using a sparger in the bottom of the jar. A water trap was placed between the pump which was insufflating air and the bioreactor in order to humidify the incoming air and reduce the evaporation losses in the reactor. The foam formation during fermentation was controlled by adding 1 mL Antifoam A (Fluka Analytical—Sigma-Aldrich Chemie GmbH, Steinheim, Germany) to the reactor whenever it was necessary (the total maximum amount of antifoam added was 6 mL). Samples were taken and analyzed every 24 h. The fermentation was replicated four times (*n* = 4).

Other technical details about the reactor can be found in Figure S1 (Supplementary Material).

### 2.5. Cell Immobilization: Repeated Batch Fermentation in the Bioreactor

**Figure 1:**
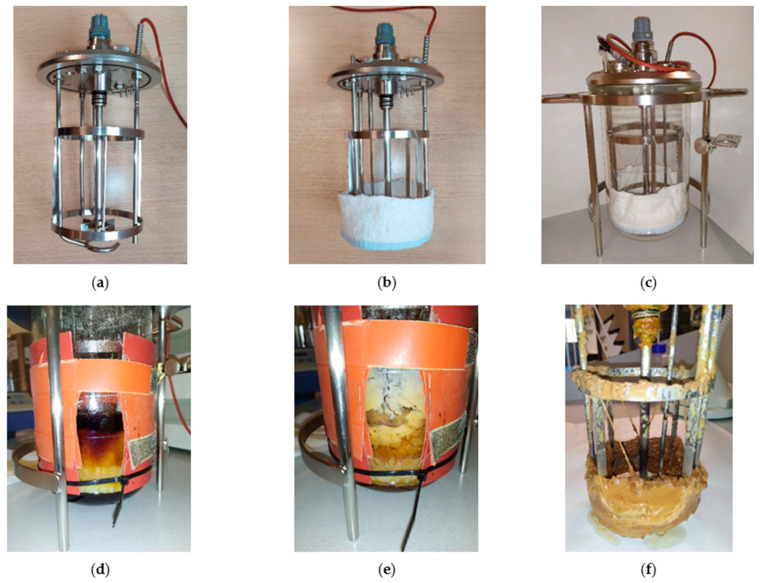
Immobilization process of *M. pollinis* MUCL 40570 on a cotton towel. (**a**) Rotor, baffle, sparger and lid of the reactor. (**b**) Attachment of the cotton towel to the baffle. (**c**) Assembled reactor. (**d**) Addition of grape must and fungal inoculation at the beginning of batch 0 (t = 0 h). (**e**) Biofilm development on the cotton towel at the end of batch 0 (t = 120 h). (**f**) Appearance of the cotton towel at the end of the experiment in batch 4 (t = 576 h post-inoculation).

A 6-cm-wide cotton towel was attached all around the bottom part of the baffle of the reactor to provide an immobilization support for the microorganisms (Figure 1a–c). The whole reactor (containing the cotton towel) was autoclaved at 121 °C for 15 min, and then, 1.2 L of sterilized grape must (with 6.88 g/L yeast extract and pH of 5.5) was fed into the reactor. The system was inoculated with 3% (*v*/*v*) of *M. pollinis* MUCL 40570 (the initial cell concentration in the bioreactor was 1.71 × 10^6^ cells/mL), and the fermentation was performed under the same temperature, agitation and aeration conditions that are described in Section 2.4. This initial batch (batch 0) lasted 120 h, and its only purpose was the colonization of the cotton towel by the fungus (Figure 1d,e).

After the end of batch 0, the exhausted broth was drawn out of the reactor using a peristaltic pump (PR 2003, JP Selecta S.A., Abrera, Spain), and it was replaced by 1.2 L of sterilized grape must (with 6.88 g/L yeast extract and pH of 5.5). The fermentation was restarted in this way during four consecutive batches (batches 1–4) of 96–120 h, whose erythritol production, sugar consumption and fungal biomass in the liquid broth were monitored daily. There was no need to inoculate the new microorganisms in batches 1–4 because the towel acted as a fungal reservoir after the end of batch 0. The cumulative duration of the four immobilization batches was 456 h (batches 1–4). In all of the cases, the foam formation was controlled by the regular addition of an antifoam agent (about 6 mL total in each batch).

### 2.6. Analytical Methods

The glucose, fructose, erythritol, ethanol, glycerol and mannitol concentrations in fermentation broths were analyzed by liquid chromatography using Agilent 1200 HPLC equipment (Agilent Technologies, Santa Clara, CA, USA) which was provided with a precolumn Micro-Guard Carbo C (Biorad, Hercules, CA, USA), a column Aminex HPX-87C (Biorad) and a refractive index detector (RID) G1362A (Agilent Technologies), and these were used according to a previously described method [16]. Briefly, the liquid samples were filtered through a nylon syringe filter with 0.20 μm pore (Agilent Technologies), and a volume of 20 μL was injected into the HPLC-RID. The column temperature was set at 80 °C, whereas the RID temperature was set at 55 °C.

The fungal cell density in the inocula and fermentation broths was measured with a Bürker chamber (Paul Marienfeld GmbH & Co. KG; Lauda-Königshofen, Germany) using a phase-contrast optical microscope Leica DM750 (Leica Microsystemic SLU, L’Hospitalet de Llobregat, Spain).

The initial and final volumes of the fermentation broth were measured, both in the flask experiments and in the bioreactor, to consider the evaporation losses in the calculations of the fermentation parameters. The levels of glucose consumption (ΔG, %), fructose consumption (ΔF, %) and total sugar consumption (ΔS, %) were calculated based on the consumed mass in relation to the initial mass [16]. The erythritol yield (Y_ERY_, g/g), glycerol yield (Y_GLY_, g/g), mannitol yield (Y_MAN_, g/g) and ethanol yield (Y_ETH_, g/g) were calculated as the ratio between the produced mass of each metabolite and the mass of total sugars consumed [16].

### 2.7. Statistical Analysis

The statistical tests (one-way ANOVA and Tukey’s HSD test) and the curve fitting (*linear model—lm* tool for polynomials) were performed and the graphs were produced with the software RStudio version 2021.09.2 Build 382—Ghost Orchid. The criteria for the curve fitting selection were based on maximizing the R-square values while minimizing the residual standard error (RSE) values.

## 3. Results and Discussion

### 3.1. Optimization of Strain and Nutrient Conditions in Flask Experiments: Free-Cell Fermentation

**Figure 2:**
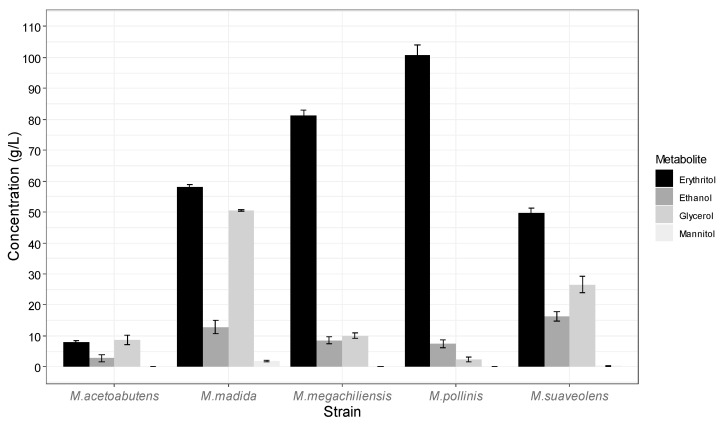
Strain comparison for the fermentation of grape must in flask experiments after 120 h (*n* = 3). Nitrogen source: 6.7 g/L yeast extract. Averages and standard deviations are shown.

The five microorganisms tested in the present work are common erythritol-producing species of *Moniliella*, which is a genus that is known for its ability to transform sugars into this polyol [7,12]. Regarding the performance of these fungi when they were fed with grape must, there were significant differences (*p* < 0.05; Tukey’s HSD test) in the erythritol production tests among the five compared strains (Figure 2; Table S2). The erythritol concentrations decreased in the following order: *M. pollinis* MUCL 40570 > *M. megachiliensis* CBS 567.85 > *M. madida* CBS 240.79 > *M. suaveolens* DSM 2552 > *M. acetoabutens* DSM 3551 (Figure 2). Regarding the other metabolites, the glycerol and ethanol production rates were remarkably higher (*p* < 0.05) for *M. madida* and *M. suaveolens* (approximately in the range of 26–51 g/L glycerol, and 13–16 g/L ethanol) than they were with the other strains, whose concentrations were in the ranges of 2–11 g/L glycerol and 2–9 g/L ethanol (Table S2). The rate of mannitol production was below 2 g/L for all of the tested fungal species. The values of glucose consumption were at their total for all of the species (Table S2). However, fructose was not as easily utilized by all of the fungi. In fact, *M. pollinis*, *M. madida* and *M. suaveolens* consumed significantly (*p* < 0.05) more fructose (97–99%) than *M. megachiliensis* did (91%), and the latter, in turn, consumed more fructose than *M. acetoabutens* did(33%). According to these results, *Moniliella pollinis* MUCL 40570 seems to be the most adequate strain for erythritol production from grape must as it produced 100.79 ± 3.35 g/L erythritol in 120 h (with a yield of 0.359 ± 0.008 g/g), with a very low formation of secondary metabolites and an almost total value of sugar consumption (98.51 ± 0.31%) (Table S2). Therefore, this microorganism was selected to perform the remaining experiments.

**Figure 3:**
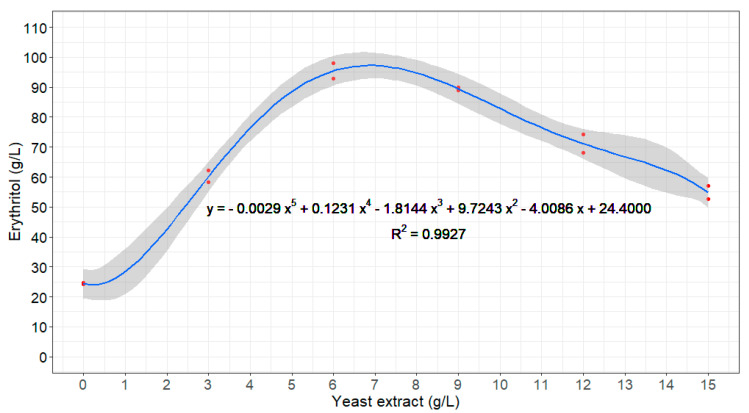
Effect of nitrogen dose (yeast extract) on erythritol production from grape must with *M. pollinis* MUCL 40570 after 120 h in flask experiments (*n* = 2). Note: Experimental observations are represented by red dots.

In terms of the erythritol dependence on the C/N ratio, it was observed that the yeast extract dosage versus the erythritol concentration could be fitted to a fifth-order polynomial equation with a maximum at 6.88 g/L yeast extract and an estimated erythritol response of 97.34 g/L (Figure 3). These optimal conditions were verified experimentally with a new fermentation batch, reaching an erythritol concentration of 107.41 ± 1.20 g/L with *M. pollinis* MUCL 40570 in 120 h, with a yield of 0.388 ± 0.011 g/g and a 99.16 ± 0.21% total sugar consumption (100 ± 0% for glucose and 98.37 ± 0.42% for fructose), as depicted in Figure S2. Consequently, a yeast extract concentration of 6.88 g/L (equivalent to 0.76 g/L TN) was set for grape must fermentation.

Concerning the partial or total replacement of the yeast extract with ammonium sulphate (nitrogen sources), it was noted that there were no significant differences (*p* > 0.05; Tukey’s HSD test) among the treatments with 100, 80, 60 and 40% yeast extract for the erythritol concentrations and yields, cell density or sugar consumption levels (Table S3). As the yeast extract doses went below 40% (i.e., when ammonium sulphate was the predominant nitrogen source), the glycerol concentrations rose, and the rate of sugar consumption decreased (Table S3). Accordingly, a mixture of 40% yeast extract and 60% ammonium sulphate would enable an important reduction of the reagent costs, yet it would lead to successful erythritol synthesis (89.73 ± 0.02 g/L erythritol, 98.77 ± 0.31% total sugar consumption, 0.313 ± 0.004 g/g erythritol yield), which is an economic advantage for future biorefineries based on agri-food by-products or surpluses such as grape must. Nevertheless, for the purposes of simplification, 100% yeast extract was employed in the ensuing bioreactor experiments.

Further details about all of the optimization experiments in flasks can be found in the Supplementary Material.

### 3.2. Free-Cell Fermentation in the Bioreactor

The free-cell fermentation of the grape must in the reactor led to the production of 41.88 ± 5.18 g/L erythritol after 96 h, with a yield of 0.17 ± 0.01 g/g (Table 2). This concentration was employed as a reference value for the subsequent immobilization tests.

Although the theoretical yield for erythritol production from hexoses is 0.678 g/g and the experimental values for small-scale systems treating pure glucose are in the range of 0.456–0.497 g/g, the reported erythritol yields for carbohydrate-rich wastes and by-products barely reached 0.12–0.38 g/g [16]. Therefore, the results obtained with the grape must lie within the expected yield values.

### 3.3. Cell Immobilization in the Reactor

**Figure 4:**
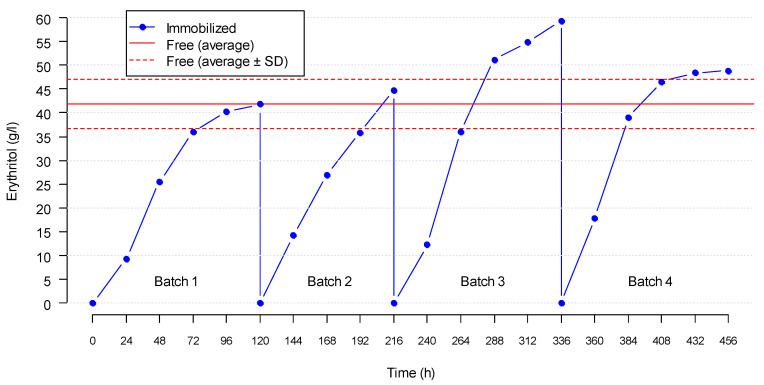
Evolution of erythritol production in the bioreactor with immobilized cells during four consecutive batches. Note: The horizontal lines represent the reference value with free cells (average ± standard deviation; 41.88 ± 5.18 g/L g/L erythritol).

Regarding the cell immobilization process on the cotton towel, fermentation happened successfully in the four consecutive batches (batches 1–4), as shown in Figure 4. According to the results, it was considered that the fermentations were finished at 96 h because the increase in the erythritol concentration at 120 h was negligible since water evaporation took place at a rate of ~6% (V_i_) on each day. The average erythritol concentration in the immobilized system after 96 h was 47.03 ± 6.16 g/L for batches 1–4 (Table 2). This concentration was similar to the reference value obtained with the free cells (41.88 ± 5.18 g/L g/L erythritol), which indicates that the immobilization technique worked properly. In fact, there were no significant differences (*p* ≫ 0.05; Tukey’s HSD test) between the free-cell and immobilized-cell fermentations for any of the tested variables shown in Table 2. Furthermore, the immobilization system proved to be efficient for at least four fermentation cycles (456 h).

The biofilm attached to the cotton towel (Figure 1f) acted as a source of microorganisms, which colonized the liquid medium (i.e., the grape must) whenever a new fermentation batch started, thus, enabling the transformation of glucose and fructose into erythritol. During the immobilization experiments in the bioreactor, the microbial cell densities at the end of the exponential growth phase attained 0.50–1.75 × 10^8^ cells/mL in the liquid broth, which was similar to the cell densities registered for the free-cell fermentations (Figure S3).

The erythritol values observed in these bioreactor experiments (~45 g/L erythritol with free or immobilized cells) are lower than those reported for the free-cell fermentations of the grape must in the Erlenmeyer flasks with smaller sample volumes (reaching about 90 g/L erythritol) (see Section 3.1). This is probably due to the differences in the agitation conditions between the bioreactor and the flasks because oxygenation is a primary factor affecting the erythritol synthesis in fungi [7,27]. Therefore, the optimization of the aeration rate and shaking in the bioreactor should be envisaged in future works in order to improve the erythritol production.

The dose of yeast extract suggested in this work is valid for this grape must sample, but the C/N ratios should be optimized for each particular case. *Moniliella* can produce erythritol in the presence of various nitrogen sources, such as yeast extract, corn steep liquor, urea, malt extract and ammonium sulphate [7,12,13,14]. Although yeast extract is an excellent source of nitrogen and vitamins for many microbial processes [28,29], it is also a costly reagent, whose estimated price for industries in Spain is EUR 7/kg (~7 $/kg) [30]. However, the process costs could be reduced remarkably if cheaper nitrogen sources were employed. According to our results, a mixture of 60% (NH_4_)_2_SO_4_ and 40% yeast extract could provide good erythritol yields from the grape must (see Section 3.1). Ammonium sulphate is an allowed food additive in the European Union (Regulation (EC) No 1333/2008), whose price is about USD 100–300 /t, which makes it an attractive nitrogen source for this process.

Erythritol is an economically attractive bioproduct that can be used in the food and pharmaceutical industries [1], and its future development as platform chemical cannot be discarded [5]. Additionally, the erythritol global market is expected to grow in the next years [31], and this is a fact which could encourage manufacturers to apply the best available techniques to their operation processes in order to increase their productivity. Consequently, the immobilization systems might become an excellent method to reduce costs in the stage of microbial cultivation.

## 4. Conclusions

The efficient cell immobilization system described herein constitutes an interesting alternative for erythritol production as it eliminates the need for continuous fungal cultivation (inocula preparation) before each fermentation batch, thus, reducing the costs of the reagents and energy. In this work, the immobilization was performed on a cotton cloth, but other immobilization supports or mechanisms could be explored in the future.

The proposed erythritol production method worked seamlessly over four fermentation cycles in a 2-L bioreactor. Nevertheless, the process needs to be ameliorated before it can be conducted at an industrial scale, especially in terms of aeration and agitation with the aim of reaching erythritol concentrations of about 90–100 g/L.

Although the system has been tested with grape must as a feedstock, it is expected to work properly when it is fed with other appropriate substrates that are rich in sugars or glycerol, regardless of the nature of the carbon source (pure or byproduct-related ones). In any case, as previously mentioned, the preferential use of by-products over pure substrates produces monetary savings. In addition, the establishment of biorefineries using agri-food by-products or surplus products as substrates (such as grape must) could revitalize the depressed rural areas where these feedstocks are generated from agricultural activities, thus, bringing additional income to farmers.

## Figures and Tables

**Table 1:** Different combinations of nitrogen sources for grape must fermentation with *M. pollinis* MUCL 40570 in flask experiments. Total nitrogen equals 0.76 g/L in all of the cases.

Experiment	Yeast Extract (%)	(NH_4_)_2_SO_4_ (%)	Yeast Extract (g/L)	(NH_4_)_2_SO_4_ (g/L)
1	100	0	6.88	0
2	80	20	5.50	0.71
3	60	40	4.13	1.43
4	40	60	2.75	2.14
5	20	80	1.38	2.86
6	0	100	0	3.57

**Table 2:** Fermentation parameters after 96 h for free cells (*n* = 4) and immobilized cells (4 consecutive batches) in the bioreactor. The feedstock was grape must that was supplemented with 6.88 g/L yeast extract, and the fungus *M. pollinis* MUCL 40570 was used. Averages and standard deviations are shown.

Type	C_X_ (× 10^8^ cells/mL) *	C_ETH_ (g/L)	C_ERY_ (g/L)	C_GLY_ (g/L)	ΔG (%)	ΔF (%)	ΔS (%)	Y_ETH_ (g/g)	Y_ERY_ (g/g)	Y_GLY_ (g/g)	Evaporation (%, V_i_)
Free	1.18 ± 0.67	4.84 ± 3.87	41.88 ± 5.18	9.16 ± 4.21	95.67 ± 3.24	88.02 ± 7.73	91.72 ± 5.27	0.02 ± 0.02	0.17 ± 0.01	0.04 ± 0.02	22.05 ± 5.26
Immobilized	0.97 ± 0.57	10.37 ± 9.38	47.03 ± 6.16	13.27 ± 1.89	96.97 ± 2.60	91.54 ± 4.42	94.27 ± 3.20	0.04 ± 0.04	0.18 ± 0.04	0.05 ± 0.01	25.18 ± 3.18

* C_X_: cell density in the liquid medium; C_ETH_: ethanol concentration; C_ERY_: erythritol concentration; C_GLY_: glycerol concentration; ΔG: glucose consumption; ΔF: fructose consumption; ΔS: total sugar consumption; Y_ETH_: ethanol yield; Y_ERY_: erythritol yield; Y_GLY_: glycerol yield; V_i_: initial volume (1.2 L).

## Data Availability

All data generated or analyzed during this study are included in this published article and its Supplementary Material files.

## Supplementary Materials

The following supporting information can be downloaded at: https://www.mdpi.com/article/10.3390/jof8121286/s1. The Supplementary Material contains information about: the initial sugar concentrations in the fermentation broths (Table S1); technical details of the bioreactor (Figure 1); a strain comparison of the flask experiments (Table S2); the evolution of fermentation under optimized nitrogen dosing (yeast extract) in the flask experiments (Figure S2); fermentation parameters with different combinations of yeast extract and ammonium sulphate (Table S3); the cell density in the liquid medium of the bioreactor (Figure S3).
