# Supplementary material for: Cell Immobilization for Erythritol Production
Source: J Fungi (Basel). 2022 Dec 8;8(12):1286. doi: 10.3390/jof8121286 (PMC9785647; doi:10.3390/jof8121286)
Supplement: Supplementary file 1 [file jof-08-01286-s001.zip › jof-2052422-supplementary.pdf]

Table S1. Initial sugar concentrations of fermentation broths (immediately after inoculation) for each experiment. Note: Broths contain grape must (carbon source), yeast extract or ammonium sulphate (nitrogen source) and microorganisms.

Experiment	Glucose (g/L)	Fructose (g/L)	Total Sugars (g/L)
Strain comparison (in flasks)	118.95	121.20	240.15
Adjustment of yeast extract dose (in flasks)	119.81	113.99	233.8
Combinations of yeast extract and ammonium sulphate (in flasks)	116.94	126.33	243.27
Free-cell fermentation (bioreactor), $n = 4$	114.36 ± 2.59	122.02 ± 4.83	236.38 ± 7.12
Immobilized fermentation (bioreactor), $n = 4$	114.39 ± 7.27	119.34 ± 10.85	233.73 ± 18.10

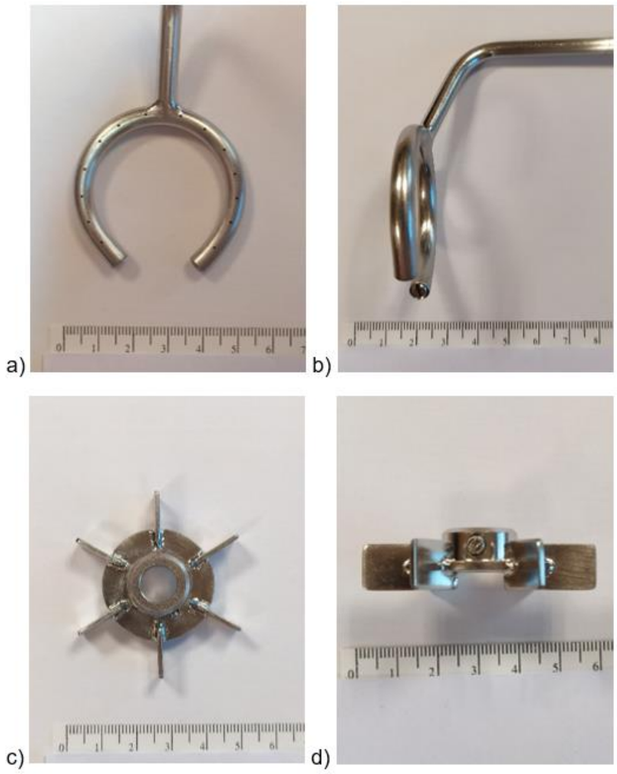

Figure S1. Photographs of (**a, b**) the sparger and (**c, d**) the rotor blades of the bioreactor. Note: The ruler shows centimeters.

Table S2. Fermentation parameters of grape must with five different fungal strains in flask experiments after 120 h (average ± standard deviation; *n* = 3). Nitrogen source: 6.7 g/L yeast extract. Note: For each column, letters between parentheses (a, b, c, d) indicate the existence of statistical differences ($p < 0.05$; Tukey HSD test) among strains; if two strains share the same letter, there are no significant differences between them for that parameter.

Strain	C_X (x 10^8 cells/mL)	C_{ETH} (g/L)	C_{ERY} (g/L)	C_{GLY} (g/L)	C_{MAN} (g/L)	ΔG (%)	ΔF (%)	ΔS (%)	Y_{ETH} (g/g)	Y_{ERY} (g/g)	Y_{GLY} (g/g)	Y_{MAN} (g/g)
M. acetoabutens DSM 3551	4.38 ± 0.49 (ab)	2.74 ± 1.15 (c)	7.85 ± 0.54 (d)	8.77 ± 1.55 (c)	0 ± 0 (a)	100 ± 0 (a)	32.66 ± 1.91 (c)	66.02 ± 0.96 (c)	1.45×10^{-2} ± 5.92×10^{-3} (c)	4.16×10^{-2} ± 2.39×10^{-3} (d)	4.65×10^{-2} ± 8.47×10^{-3} (c)	0 ± 0 (a)
M. madida CBS 240.79	5.11 ± 0.59 (b)	12.87 ± 2.04 (b)	58.05 ± 0.81 (b)	50.50 ± 0.20 (b)	1.74 ± 0.24 (b)	100 ± 0 (a)	99.05 ± 0.06 (a)	99.52 ± 0.03 (a)	4.46×10^{-2} ± 6.92×10^{-3} (bd)	2.01×10^{-1} ± 4.12×10^{-3} (b)	1.75×10^{-1} ± 1.99×10^{-3} (b)	6.04×10^{-3} ± 7.86×10^{-4} (b)
M. megachi-liensis CBS 567.85	7.63 ± 0.96 (c)	8.54 ± 1.24 (a)	81.10 ± 1.92 (c)	10.09 ± 0.91 (c)	0 ± 0 (a)	100 ± 0 (a)	90.87 ± 0.60 (b)	95.39 ± 0.30 (b)	3.16×10^{-2} ± 4.53×10^{-3} (ab)	3.00×10^{-1} ± 5.89×10^{-3} (c)	3.73×10^{-2} ± 3.35×10^{-3} (c)	0 ± 0 (a)
M. pollinis MUCL 40570	2.80 ± 0.11 (a)	7.48 ± 1.20 (a)	100.79 ± 3.35 (a)	2.44 ± 0.78 (a)	0 ± 0 (a)	100 ± 0 (a)	97.04 ± 0.62 (a)	98.51 ± 0.31 (a)	2.66×10^{-2} ± 3.96×10^{-3} (ac)	3.59×10^{-1} ± 7.50×10^{-3} (a)	8.72×10^{-3} ± 2.88×10^{-3} (a)	0 ± 0 (a)
M. suaveolens var. *nigra* DSM 2552	3.58 ± 0.63 (ab)	16.25 ± 1.45 (b)	49.85 ± 1.42 (e)	26.55 ± 2.67 (d)	0.23 ± 0.20 (a)	100 ± 0 (a)	98.85 ± 0.25 (a)	99.42 ± 0.12 (a)	5.60×10^{-2} ± 4.94×10^{-3} (d)	1.72×10^{-1} ± 5.02×10^{-3} (e)	9.16x10-2 ± 9.49×10^{-3} (d)	7.83×10^{-4} ± 6.79×10^{-4} (a)

C_X: cell density in the liquid medium; C_{ETH}: ethanol concentration; C_{ERY}: erythritol concentration; C_{GLY}: glycerol concentration; C_{MAN}: mannitol concentration; ΔG: glucose consumption, ΔF: fructose consumption, ΔS: total sugar consumption, Y_{ETH}: ethanol yield, Y_{ERY}: erythritol yield, Y_{GLY}: glycerol yield; Y_{MAN}: mannitol yield.

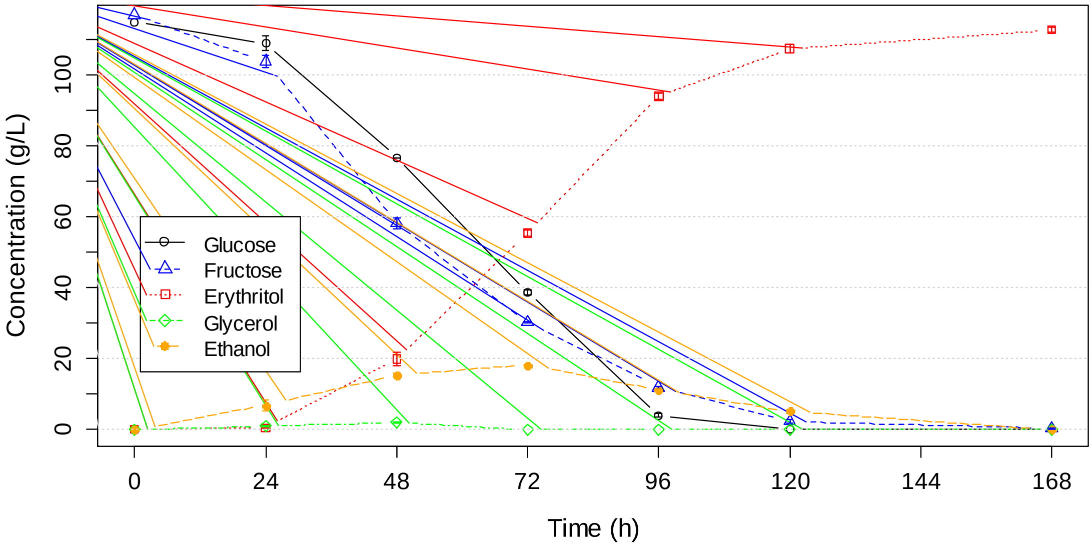

Figure S2. Evolution of grape must fermentation with *M. pollinis* MUCL 40570 in flask experiments under optimized nitrogen dosing (6.88 g/L yeast extract; equivalent to 0.76 g/L TN). Average values ± standard deviations are shown (n = 2).

Table S3. Fermentation parameters (120 h) for grape must in flask experiments with different combinations of yeast extract and ammonium sulphate as nitrogen sources (as described in Table 1 in the manuscript), using *M. pollinis* MUCL 40570 (average ± standard deviation; n = 2). Note: For each column, letters between parentheses (**a, b, c, d**) indicate the existence of statistical differences ($p < 0.05$; Tukey HSD test) among treatments; if two treatments share the same letter, there are no significant differences between them for that parameter.

Yeast extract (%)	Cell Density (cells/mL) x 10^8	C_{ETH} (g/L)	C_{ERY} (g/L)	C_{GLY} (g/L)	ΔG (%)	ΔF (%)	ΔS (%)	Y_{ETH} (g/g)	Y_{ERY} (g/g)	Y_{GLY} (g/g)
100	2.69 ± 0.31 (a)	5.62 ± 2.35 (a)	89.12 ± 0.50 (a)	1.29 ± 0.16 (d)	100 ± 0 (a)	99.09 ± 0.39 (a)	99.53 ± 0.20 (a)	1.95x10^{-2} ± 7.96x10^{-3} (a)	3.10x10^{-1} ± 5.29x10^{-3} (a)	4.47x10^{-3} ± 6.16x10^{-4} (d)
80	2.34 ± 0.22 (a)	5.27 ± 1.96 (a)	89.93 ± 1.19 (a)	0.79 ± 0.14 (d)	100 ± 0 (a)	99.49 ± 0.06 (a)	99.74 ± 0.03 (a)	1.83x10^{-2} ± 6.76x10^{-3} (a)	3.10x10^{-1} ± 5.29x10^{-3} (a)	2.74x10^{-3} ± 4.86x10^{-4} (d)
60	2.14 ± 0.07 (a)	6.91 ± 3.05 (a)	90.17 ± 0.49 (a)	1.54 ± 0.19 (d)	100 ± 0 (a)	98.94 ± 0.29 (a)	99.45 ± 0.15 (a)	2.37x10^{-2} ± 1.06x10^{-2} (a)	3.09x10^{-1} ± 2.40x10^{-6} (a)	5.25x10^{-3} ± 6.25x10^{-4} (d)
40	2.13 ± 0.31 (a)	4.86 ± 1.91 (a)	89.73 ± 0.02 (a)	2.85 ± 0.49 (c)	100 ± 0 (a)	97.63 ± 0.60 (a)	98.77 ± 0.31 (a)	1.69x10^{-2} ± 6.47x10^{-3} (a)	3.13x10^{-1} ± 3.54x10^{-3} (a)	9.94x10^{-3} ± 1.83x10^{-3} (c)
20	1.80 ± 0.11 (a)	4.52 ± 0.47 (a)	78.67 ± 2.54 (b)	4.10 ± 0.05 (b)	99.88 ± 0.10 (a)	93.36 ± 1.27 (b)	96.49 ± 0.71 (b)	1.63x10^{-2} ± 1.84x10^{-3} (a)	2.83x10^{-1} ± 6.76x10^{-3} (b)	1.47x10^{-2} ± 3.02x10^{-4} (b)
0	2.41 ± 0.44 (a)	0 ± 0 (a)	62.69 ± 0.09 (c)	7.84 ± 0.09 (a)	94.62 ± 0.28 (b)	83.37 ± 0.97 (c)	88.78 ± 0.37 (c)	0 ± 0 (a)	2.41x10^{-1} ± 4.64x10^{-4} (c)	3.01x10^{-2} ± 2.51x10^{-4} (a)

C_{ETH}: ethanol concentration; C_{ERY}: erythritol concentration; C_{GLY}: glycerol concentration; ΔG: glucose consumption, ΔF: fructose consumption, ΔS: total sugar consumption, Y_{ETH}: ethanol yield, Y_{ERY}: erythritol yield, Y_{GLY}: glycerol yield.

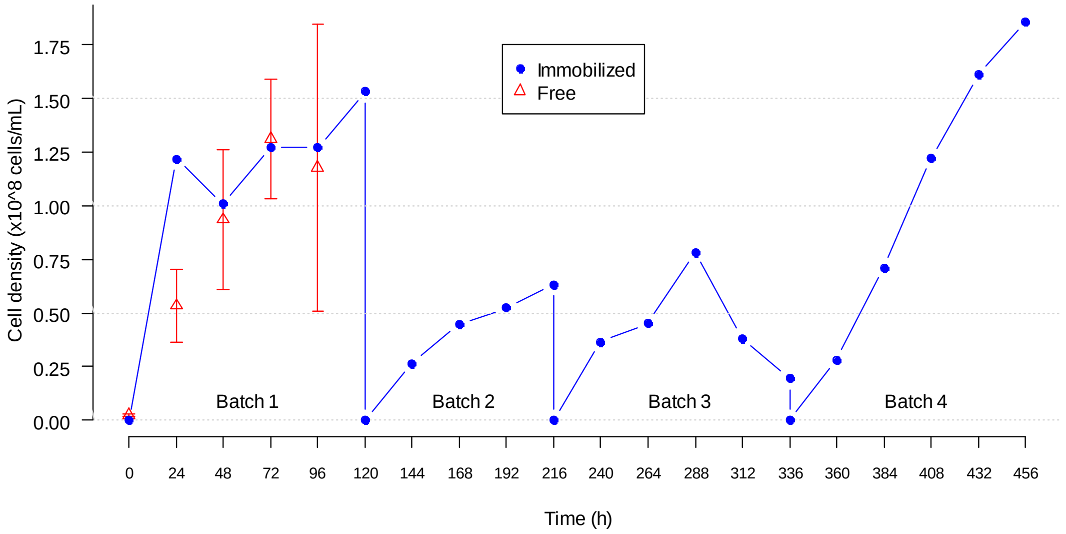

Figure S3. Cell density in the liquid medium of the bioreactor during the four consecutive immobilization batches (filled circles). The values of the free-cell fermentation are shown for comparison (empty triangles).
